# Hypophosphatemic rickets: diagnosis and treatment

**DOI:** 10.20945/2359-4292-2026-0044

**Published:** 2026-04-01

**Authors:** Hailey Bruneau, Clemens Bergwitz

**Affiliations:** 1 Yale School of Medicine, Section Endocrinology, Department of Medicine, New Haven, CT, USA

**Keywords:** Burosumab, C-type natriuretic peptide, fibroblast growth factor 23, hereditary hypophosphatemic rickets with hypercalcuria, X-linked hypophosphatemia

## Abstract

Hypophosphatemicrickets (HR) represents a heterogeneous group of disorders characterized by renal phosphate wasting, impaired bone mineralization, and skeletal deformities. This narrative review provides an overview of phosphate homeostasis and the molecular mechanisms underlying HR, focusing on the role of Fibroblast Growth Factor 23 (FGF23) in regulating renal phosphate use and vitamin D metabolism. The clinical, biochemical, and genetic features of both FGF23-dependent and -independent forms of HR are discussed, including X-linked hypophosphatemia (XLH), autosomal dominant hypophosphatemic rickets (ADHR), autosomal recessive hypophosphatemic rickets (ARHR), tumor-induced osteomalacia (TIO), hereditary hypophosphatemic rickets with hypercalciuria (HHRH), and Fanconi syndrome. Advances in understanding these mechanisms have led to the development of targeted therapies, such as burosumab, which are redefining the clinical management of affected individuals.

## INTRODUCTION

Phosphate is a crucial mineral for skeletal development and metabolic health, playing a role in bone mineralization, cellular energy metabolism, and enzymatic activity ^(1,2)^. Phosphate homeostasis is maintained through a tightly regulated relationship between intestinal absorption ^(3)^, renal reabsorption, and storage as hydroxyapatite by bone remodeling ^(4,5)^. Fibroblast growth factor 23 (FGF23) acts as a key regulator ^(6,7)^. Disruption of this balance leads to hypoand hyperphosphatemic disorders, as reviewed by us ^(8)^ and others ^(9,10)^. The focus of this review will be on hypophosphatemic rickets (HR), a group of disorders characterized by impaired bone mineralization due to impaired phosphate availability. HR disorders can be divided into disorders of phosphate absorption from the diet in the gut and due to chronic renal phosphate wasting, which in turn can be FGF23-dependent, Parathyroid Hormone (PTH)-dependent, and FGF23/PTH-independent, each with specific genetic and pathophysiological mechanisms ^(7,11,12)^. Differentiating HR subtypes is important for targeted and effective management strategies. This manuscript is structured as a narrative review. Rather than employing a formal systematic review of methodology, we conducted a focused, non-systematic synthesis of the literature to integrate novel mechanistic, clinical, and therapeutic insights across the spectrum of hypophosphatemic disorders. Emphasis was placed on studies elucidating hormonal regulation, genetic mechanisms, diagnostic algorithms, and emerging targeted therapies.

## PHOSPHATE HOMEOSTASIS AND THE ROLE OF FGF23

**Figure 1:**
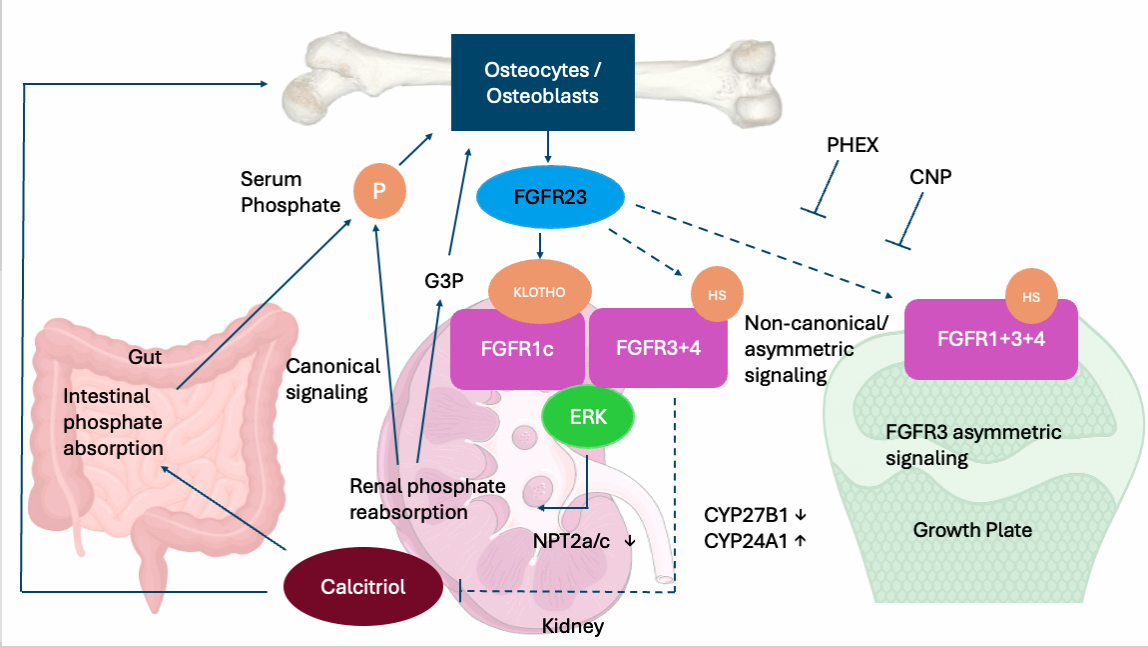
**Integrated regulation of phosphate homeostasis by FGF23 through canonical and non-canonical signaling pathways**. Osteocytes and osteoblasts secrete FGF23, which circulates to the kidney and growth plates of growing children. In the kidney, canonical signaling occurs through the formation of an FGF23-KLOTHO-FGFR1c-Heparin sulfate (HS) complex, activating ERK signaling to suppress NPT2a/NPT2c-mediated renal phosphate reabsorption. Non-canonical signaling via FGFR3 or 4 to decrease CYP27B1 while increasing CYP24A1, leads to reduced calcitriol synthesis. Lower calcitriol levels diminish intestinal phosphate absorption. FGF23 may also signal through non-canonical/asymmetric FGFR3-dependent pathways at the growth plates of growing children, which are independent of KLOTHO and modulated by HS. These pathways influence chondrocyte differentiation and may contribute to skeletal abnormalities in hypophosphatemic disorders. PHEX and C-type natriuretic peptide (CNP) act as modulators that oppose excessive FGF23 signaling in the growth plate. Serum phosphate feeds back on osteocytes to regulate FGF23 production, completing the homeostatic loop. G3P denotes glycerol-3-phosphate. Solid lines represent canonical, αKlotho-dependent endocrine signaling supported by genetic, biochemical, and structural evidence ^(20)^. Broken lines represent non-canonical, sometimes αKlotho-independent pathways ^(22)^.

Phosphate balance in the body is strictly regulated through coordinated actions in the intestine, kidney, and bone ^(13)^. FGF23 acts as a key regulator of renal phosphate excretion by inhibiting the renal sodium-dependent phosphate cotransporters NPT2a (encoded by *SLC34A1*) and NPT2c (encoded by *SLC34A3*) ^(14)^. FGF23 also suppresses the synthesis of calcitriol in the proximal tubules ^(15,16)^. Calcitriol refers to 1,25-dihydroxyvitamin D3 however, both ergocalciferol (D2) and cholecalciferol (D3) can be activated in the kidneys to the active hormone that regulates calcium ^(17)^. For the purpose of this review 1,25-dihydroxyvitamin D and calcitriol are used interchangably. By inhibiting calcitriol FGF23 reduces intestinal phosphate absorption, restoring elevated serum phosphate levels toward normal ^(18)^. Canonical action via Fibroblast growth receptor 1c (FGFR1c) requires klotho (KL) and heparan sulfate for the formation of quaternary complexes FGF23:KL:FGFR1c:HS 2:2:2:2, which is responsible for phosphate excretion in the kidneys ^(19,20)^. In addition, recent crystal structure studies suggest FGF23 may signal in a 1:2:1:1 asymmetric quaternary complex with FGFR3 or 4 ^(21)^ or even in a KL-independent manner in a tissue-specific fashion, which is responsible for non-canonical actions of FGF23 on calcitriol synthesis and cardiac remodeling ^(22)^ (**Figure 1**). PTH also inhibits NPT2a and NPT2c ^(23)^, however, because of its primary role in calcium homeostasis, it differs from FGF23 and stimulates calcitriol synthesis to restore low blood calcium levels toward normal ^(24)^. Calcitriol and phosphate, in turn, stimulate gene expression and secretion of bioactive FGF23 ^(25)^. Dietary phosphate is absorbed in the small intestine via the sodium-phosphate cotransporter NPT2b (30%) and via paracellular transport (70%) ^(3)^. Calcitriol enhances intestinal phosphate uptake by increasing the abundance of NPT2b at the apical membrane and by transcriptionally activating *SLC34A2*, the gene encoding NPT2b. This occurs through direct binding of the vitamin D receptor (VDR)/retinoid X receptor (RXR) heterodimer to vitamin D response elements within the *SLC34A2* promoter ^(26)^. Calcitriol induces expression of tight-junction components that facilitate paracellular calcium movement, thereby amplifying overall absorptive capacity ^(27)^. Conversely, paracellular transport of phosphate in the gut appears to be calcitriol-independent as discussed in the next chapter ^(28)^.

The phosphate-independent features of XLH resemble the *FGFR3* activation disorders ^(29)^, achondroplasia and hypochondroplasia and are likely caused by excess FGF23 activity at sites other than the kidney ^(22)^. Hyp mouse fetuses, a model of XLH, are not exposed to an hypophosphatemic environment *in utero*, but rather extremely high levels of FGF23 ^(30)^. The classic findings in newborn Hyp mice include craniofacial abnormalities and pelvic malformation, rather than rickets, and cannot be due to low ambient phosphate. This finding parallels the clinical observation in human children, in whom radiographic evidence of rickets (impaired mineralization of growth plate cartilage) is not evident until several months of age, after a prolonged exposure to hypophosphatemia, which is generally not apparent until several weeks of age. Conversely, their appearance at birth is similar to that of children affected with achondroplasia and hypochondroplasia; overlapping features include the head shape indicative of aberrant fusion of sutures of cranial bones, Chiari malformation, coxa vara, short stature, and prominent lumbar lordosis among others ^(8)^. Importantly, clinical studies in children and young adults with XLH have demonstrated preserved or increased bone mineral density (BMD), particularly at the lumbar spine and proximal femur ^(31)^. These findings occur early in life and cannot be attributed solely to enthesopathy or degenerative changes, which typically emerge later in adulthood. Rather, they likely reflect intrinsic alterations in bone modeling and mineralization associated with excess FGF23 signaling, aberrant FGFR3 pathway activation or PHEX deficiency ^(29,32)^. This observation further supports the concept that certain skeletal features of XLH are independent of hypophosphatemia.

Dysregulation of FGF23, whether through excessive secretion ^(6)^ or resistance to degradation ^(7)^, can lead to phosphate-wasting disorders, such as FGF23-dependent hypophosphatemic rickets. In contrast, loss of function of NPT2a and NPT2c transporters causes FGF23-independent hypophosphatemic rickets ^(12,33)^. Comprehending the molecular mechanisms of phosphate metabolism is essential for diagnosing and managing these conditions, as discussed in the following sections.

## DISORDERS OF PHOSPHATE ABSORPTION FROM THE DIET IN THE GUT

Phosphate absorption in the gastrointestinal tract occurs primarily in the small intestine, with both active transcellular transport and passive paracellular diffusion contributing to uptake ^(3)^. Approximately 30% of dietary phosphate is absorbed through the sodium-dependent phosphate cotransporter NPT2b (*SLC34A2*) located on the apical membrane of enterocytes in the jejunum and ileum ^(3)^. NPT2b activity is strongly regulated by calcitriol, which enhances transporter expression and increases active phosphate uptake ^(17)^.

Most phosphate absorption occurs via paracellular transport (~70%), which is dependent on electrochemical gradients and tight junction proteins, such as claudins ^(28)^. Sodium/hydrogen exchanger 3 (NHE3) indirectly contributes by modulating luminal pH and sodium flux, which influence phosphate permeability ^(28,34)^. Experimental data show that this pathway can be inhibited pharmacologically, such as Tenapanor, an NHE3 inhibitor developed for irritable bowel syndrome with constipation, which reduces intestinal phosphate uptake by decreasing paracellular phosphate permeability. It has also received approval in Canada and the United States for the management of hyperphosphatemia in patients with chronic kidney disease undergoing dialysis ^(28)^.

Several dietary and hormonal factors regulate intestinal phosphate absorption. Calcitriol is the primary stimulator, upregulating NPT2b and enhancing overall uptake ^(3,17)^. FGF23 indirectly reduces phosphate absorption by suppressing renal production of calcitriol, therefore decreasing NPT2b expression ^(15)^. Likewise, parathyroid hormone indirectly stimulates gut phosphate transport through it’s positive regulation of calcitriol synthesis ^(35)^.

## GENETIC DISORDERS OF INTESTINAL PHOSPHATE ABSORPTION

Primary genetic causes of impaired intestinal phosphate absorption are rare but provide insight into phosphate physiology. Loss-of-function variants in *SLC34A2* (which encodes NPT2b) in humans cause pulmonary alveolar microlithiasis, but intestinal phosphate malabsorption does not appear to be a significant problem in these individuals ^(36)^. Animal studies, however, suggest that NPT2b ablation in the intestine alters systemic phosphate balance, especially under conditions of high dietary phosphate intake ^(3)^.

## ACQUIRED CAUSES OF IMPAIRED PHOSPHATE ABSORPTION

Acquired conditions are more commonly responsible for impaired intestinal phosphate absorption and can significantly contribute to hypophosphatemia. Vitamin D deficiency is the most frequent cause, as it diminishes 25-hydroxyvitamin D (25-OHD) levels, the substrate for calcitriol production, and as a result impairs active intestinal phosphate uptake ^(37)^. Importantly, calcitriol levels are not invariably low in vitamin D deficiency; they may be normal or even transiently elevated due to secondary hyperparathyroidism stimulating renal 1α-hydroxylase activity ^(38)^, particularly in early or moderate deficiency states. This mechanism is particularly relevant in nutritional rickets and may clinically overlap with hereditary HR. Dietary factors can also reduce intestinal phosphate availability, including excessive intake of phosphate binders such as calcium, sevelamer, or lanthanum ^(39,40)^, as well as diets high in phytate-rich foods like whole grains or legumes, which contain poorly absorbable organic phosphate ^(41)^. Several medications interfere with intestinal phosphate handling. For example, niacin therapy has been reported to reduce phosphate absorption by blocking Npt2b ^(42)^, while the NHE3 inhibitor Tenapanor lowers paracellular phosphate permeability ^(28,34)^. In addition, infants receiving specialized elemental formulas such as Neocate™ have been shown to develop phosphate deficiency and hypophosphatemic rickets, likely due to reduced intestinal phosphate bioavailability inherent to these preparations ^(43)^. Recognition of this complication has prompted reformulations of Neocate™.

## CLINICAL IMPLICATIONS

Although disorders of intestinal phosphate absorption are less common than renal phosphate wasting syndromes, they are an important consideration in the differential diagnosis of hypophosphatemic rickets. Key distinguishing features include low urinary phosphate excretion (reflecting intact renal conservation) and improvement with dietary supplementation or vitamin D repletion ^(38)^. In practice, patients with vitamin D deficiency, malabsorption, or exclusive elemental formula feeding should be carefully evaluated for concurrent phosphate deficiency to avoid misdiagnosis as renal HR ^(43)^.

## DISORDERS OF RENAL PHOSPHATE WASTING

For clinical diagnostic purposes, these disorders are best classified into FGF23-dependent, PTH-dependent, and FGF23/PTH-independent disorders ^(6,12)^. Because PTH-dependent phosphate wasting disorders are primarily calcium disorders, we will focus in this review on FGF23-dependent and FGF23/PTH-independent renal phosphate wasting disorders. PTH-dependent hypophosphatemic states include primary hyperparathyroidism, secondary hyperparathyroidism and tertiary hyperparathyroidism, which may be due to vitamin D deficiency or chronic kidney disease ^(44)^, in which elevated PTH promotes renal phosphate wasting via downregulation of proximal tubular phosphate transport ^(45)^.

## FGF23-DEPENDENT HYPOPHOSPHATEMIC RICKETS

FGF23-dependent hypophosphatemic rickets refers to a group of disorders characterized by overproduction or reduced degradation of FGF23, resulting in renal phosphate wasting and reduced calcitriol levels ^(6)^. Chronic hypophosphatemia impairs bone mineralization and causes bowing and short stature in children and stress fractures in adults ^(6,7)^. The most common forms of FGF23-dependent hypophosphatemic rickets include X-linked hypophosphatemia (XLH) ^(11)^, autosomal dominant hypophosphatemic rickets (ADHR) ^(7,46)^, autosomal recessive hypophosphatemic rickets (ARHR) ^(47)^, and tumor-induced osteomalacia (TIO) ^(6)^.

### X-linked hypophosphatemia (XLH)

X-Linked Hypophosphatemia (XLH) is the most prevalent form of hereditary hypophosphatemic rickets, caused by loss-of-function variants in the *PHEX* (phosphate-regulating endopeptidase homolog X-linked) gene ^(11)^. The PHEX protein is a membrane-associated metalloprotease expressed in osteocytes and odontoblasts. Primary biochemical studies demonstrate that *PHEX* has protease activity and can cleave mineralization-inhibiting ASARM peptides, supporting a physiological enzymatic role in regulating extracellular matrix mineralization ^(48)^. Loss-of-function variants in *PHEX* are associated with increased FGF23 expression in bone and renal phosphate wasting ^(25)^ and decreased vitamin D activation ^(15)^. XLH typically presents with rickets, progressive lower limb deformities, stress fractures and short stature, which are generally directly attributed to hypophosphatemia ^(49)^. Additional features include craniosynostosis and dental abscesses during childhood ^(50)^ and premature osteoarthritis and enthesopathies in adulthood ^(51)^, the mechanism of which is less clear and may in part be mediated by non-canonical signaling of FGF23 via *FGFR3* ^(38,52)^. At the biochemical level, patients exhibit low serum phosphate, elevated FGF23 levels, normal to low calcitriol, normal or elevated alkaline phosphatase and normal or elevated parathyroid hormone (PTH) levels ^(11,25,53)^. Diagnosis is based on these biochemical markers, characteristic clinical findings, and radiographic evidence of rickets ^(54)^, and confirmed by genetic testing for *PHEX* loss-of-function variants ^(11)^.

Conventional and burosumab therapy for XLH will be discussed below; however, specific issues in the disorder include improving dental hygiene, treating dental abscesses ^(55)^, and surgical correction of deformities in children ^(37)^. Sometimes growth hormone therapy is considered ^(56)^ as discussed below. Adults often require dedicated orthopedic care for pre-mature osteoarthritis and enthesopathies ^(57)^.

### Autosomal dominant hypophosphatemic rickets (ADHR)

Another FGF23-dependent subtype is autosomal dominant hypophosphatemic rickets (ADHR), which presents with variable expressivity and often delayed onset. ADHR is caused by missense variants in *FGF23* that affect arginine residues at positions 176 or 179 within the RXXR subtilisin/furin protease recognition site ^(7)^ These variants render FGF23 resistant to intracellular proteolytic cleavage, resulting in stabilization of intact biologically active FGF23 ^(58)^. However, resistance to proteolytic processing alone is insufficient to sustain FGF23 transcription which is tightly regulated in systemic factors, including serum phosphate levels ^(59)^. Chronic hypophosphatemia normally suppresses *FGF23* gene expression, even when the encoded protein is cleavage-resistant. Therefore, it is believed that additional environmental or physiological triggers are required for manifestation in ADHR.

Iron deficiency is the most well-characterized such trigger ^(60)^. Iron deficiency increases FGF23 transcription, but in healthy individuals intracellular proteolytic cleavage prevents accumulation of intact biologically active FGF23. In FGF23 variants causing ADHR render the RXXR subtilisin, furin cleavage site resistant to proteolysis, leading to increased production and reduced degradation of intact FGF23 protein, precipitating renal phosphate wasting and symptomatic hypophosphatemia ^(61)^. The requirement for iron deficiency as trigger explains the variable penetrance and fluctuating clinical course observed in ADHR ^(62)^.

Clinically, ADHR presents with less severe extremity deformities, muscle weakness, bone pain, and dental issues ^(7)^. Biochemical abnormalities include elevated FGF23, low serum phosphate, and normal to low calcitriol ^(6)^. Diagnosis is confirmed through genetic testing ^(7)^.

Treatment strategies for ADHR are like those for XLH, as specified below. In cases of iron deficiency, iron replacement therapy has been shown to reduce FGF23 levels and improve phosphate homeostasis ^(60)^.

### Autosomal recessive hypophosphatemic rickets (ARHR)

ARHR is a rare form of hereditary rickets, caused by *DMP1* (ARHR1) ^(46)^ or *ENPP1* (ARHR2) ^(47)^ loss-of-function variants. Both genes suppress FGF23 expression by mechanism not well understood, and loss-of-function variants in these genes lead to excessive FGF23 secretion ^(59)^ and renal phosphate wasting ^(63)^. Patients exhibit skeletal deformities, short stature, and dental abnormalities, such as those seen in XLH ^(46)^.

Diagnosis is based on biochemical markers, including low phosphate, high FGF23, and low to normal calcitriol ^(46,64)^. In addition, there may be characteristic clinical features associated with ENPP1 enzyme deficiency, which causes generalized arterial calcification of infancy (GACI) and osteoporosis in adults and the diagnosis is confirmed by genetic testing ^(65)^. Treatment consists of phosphate and vitamin D supplementation. There is ongoing research on targeted therapies, such as ENPP1-Fc enzyme replacement therapy ^(66)^.

### Tumor-induced osteomalacia (TIO)

In contrast to inherited forms, tumor-induced osteomalacia (TIO) represents an acquired FGF23-dependent disorder caused by mesenchymal tumors that secrete excessive amounts of FGF23. This leads to severe phosphate wasting and osteomalacia ^(6)^. Although described in children, TIO typically presents in adulthood with progressive bone pain, muscle weakness, and bone fractures ^(67)^. Biochemically, patients exhibit profound hypophosphatemia, elevated FGF23 levels, and suppressed calcitriol ^(6)^.

Diagnosis involves measuring serum FGF23 by venous sampling and localizing the tumor with functional imaging techniques, such as 68Ga-DOTATATE PET/CT scanning ^(68)^. Genetic testing of resected tumors in 60% of cases reveals somatic genetic rearrangements of *FGF1* or *FGFR1* with the fibronectin gene (FN1) ^(69)^, and in a single case the rearrangement of *NIPBL-BEND2* ^(70)^, which are thought to drive tumor-specific overproduction of FGF23.

### Current therapy

Treatment for FGF23-dependent hypophosphatemic rickets, including XLH, ADHR, and ARHR, has historically involved oral phosphate supplementation in combination with active vitamin D analogs such as calcitriol ^(31)^. This approach supports skeletal mineralization and improves rickets and osteomalacia of affected individuals, however, it is limited by the requirement for frequent dosing and side effects, including the development of secondary hyperparathyroidism and nephrocalcinosis ^(71)^.

Over time, prolonged secondary hyperparathyroidism may progress to tertiary hyperparathyroidism, characterized by autonomous PTH secretion resulting in hypercalcemia ^(72)^.

Medical management with active vitamin D analogs, calcimimetics (e.g., cinacalcet), or adjustment of phosphate dosing may improve secondary hyperparathyroidism, however, parathyroidectomy may be required in patients with tertiary hyperparathyroidism, particularly when hypercalcemia contributes to nephrocalcinosis ^(73)^.

Burosumab, a monoclonal antibody that inhibits FGF23, represents a significant advancement in the management of these conditions ^(74)^. Approved initially for both children and adults with XLH and, more recently, for tumor-induced osteomalacia (TIO) ^(75)^, burosumab directly targets the pathophysiology of FGF23 excess, leading to improved phosphate homeostasis, linear growth, physical function, and quality of life ^(76)^. Despite its benefits, limitations include high cost, limited availability in certain countries, and lack of long-term safety data. Moreover, it is not recommended for use in patients with severe kidney dysfunction and has not been fully validated for FGF23-mediated hypophosphatemic rickets subtypes other than XLH and TIO. Therefore, treatment must be individualized, and more research is needed to expand access and optimize outcomes across all forms of FGF23-dependant hypophosphatemia. In tumor-induced osteomalacia (TIO), the standard treatment is complete surgical resection of the responsible phosphaturic mesenchymal tumor, which typically results in rapid normalization of FGF23 levels and correction of hypophosphatemia ^(67)^. In unresectable cases, conventional therapy or burosumab may be used for symptomatic management ^(76)^ and chemotherapeutic approaches are needed for rare cases with metastatic TIO ^(77)^.

Special consideration should be given to treatment during transitional periods, particularly from late childhood to adolescence and from adolescence to adulthood in patients with genetic forms of hypophosphatemic rickets. During adolescence, puberty increases skeletal growth velocity, which may exacerbate deformities and functional impairment, necessitating careful adjustment of phosphate or burosumab dosing. Transition to adult care is another critical period, as medication adherence often declines and long-term complications such as enthesopathy, osteoarthritis, dental disease, and persistent bone pain may emerge despite apparent radiographic healing. Adult treatment goals shift from promoting linear growth to optimizing functional outcomes, minimizing pain, preventing fractures, and reducing extraskeletal complications. Structured transition programs and coordinated multidisciplinary follow-up are therefore recommended to ensure continuity of care and prevent disease progression across the lifespan ^(78)^.

### Emerging therapies and future directions for FGF23-dependent HR

The development of burosumab has shown that neutralizing FGF23 can markedly improve outcomes in XLH and TIO ^(74)^. Parallel strategies could include modulation of FGF23 cleavage and stability, such as pharmacologic agents that enhance proteolysis or prevent pathological stabilization of intact FGF23, with the goal of reducing hormone activity ^(79)^. But FGF23 neutralizing therapy leaves certain aspects of the phenotype unaddressed, namely the dental, craniofacial and chondrodysplastic features of XLH and tumor recurrence in TIO ^(75)^. Several investigational approaches aim to broaden the therapeutic landscape. Selective FGFR inhibitors, such as infigratinib, are being tested for TIO by targeting signaling downstream of FGF23 ^(77)^. Trametinib is a MEK-inhibitor shown to improve hypophosphatemia in patients with cutaneous nevus syndrome ^(80)^ and it may be suitable to treat other forms of FGF23-dependant hypophosphatemia as well.

C-type natriuretic peptide (CNP) analogs like vosoritide are being evaluated for their ability to improve the craniofacial and chondrodysplasia in patients with XLH, which may also improve adult height ^(81)^.

Gene-directed approaches aimed at correcting underlying genetic defects remain in preclinical stages. For example, *PHEX* replacement or correction of protease-resistant *FGF23* variants represent long-term prospects to restore normal phosphate metabolism at the molecular level.

## FGF23-INDEPENDENT HYPOPHOSPHATEMIC RICKETS

While FGF23-dependent mechanisms are responsible for most cases of hypophosphatemic rickets, a smaller group of disorders arises from FGF23-independent defects in phosphate utilization ^(82)^. These conditions directly impair renal phosphate reabsorption or vitamin D metabolism ^(18)^.

In contrast to FGF23-dependent forms, FGF23-independent hypophosphatemic rickets is characterized by intrinsic defects in renal phosphate transport or proximal tubular function rather than excess FGF23 activity ^(64)^. The most prominent conditions include hereditary hypophosphatemic rickets with hypercalciuria (HHRH) ^(64)^, Fanconi syndrome ^(2)^, and other rare genetic causes ^(33)^.

### Hereditary hypophosphatemic rickets with hypercalciuria (HHRH)

The best-known FGF23-independent disorder is hereditary hypophosphatemic rickets with hypercalciuria (HHRH), which is characterized by high-normal or frankly elevated blood calcium levels and hypercalciuria ^(83)^. HHRH is caused by loss-of-function variants in *SLC34A3*, which encodes the NPT2c transporter in the proximal tubules of the kidney ^(64)^. Loss of NPT2c leads to isolated renal phosphate wasting, resulting in hypophosphatemia, increased proximal tubular 1-alpha hydroxylase (encoded by *CYP27B1*) and reduced 24-hydroxylase (encoded by *CYP24A1*) activity, which may be a consequence of appropriately suppressed FGF23 levels ^(18)^. This causes elevated calcitriol, suppression of PTH, enhanced intestinal and decreased distal tubular calcium absorption, and hypercalciuria. Elevated calcitriol levels are a notable difference between HHRH and FGF23-related disorders, where calcitriol levels are typically low or inappropriately normal ^(6)^.

Clinically, patients present with rickets, nephrocalcinosis, and kidney stones in the first decade of life ^(83)^. Diagnosis is based on biochemical findings (low phosphate, high calcitriol, hypercalciuria, normal or low FGF23) and confirmed by genetic testing. Treatment includes phosphate supplementation alone. Unlike FGF23-related HR, active vitamin D analogs are not indicated given the already elevated 1,25(OH)_2_D levels.

### Fanconi syndrome and other causes of phosphate wasting

Fanconi Syndrome represents a generalized dys-function of the proximal renal tubules, resulting in impaired reabsorption of multiple solutes, including phosphate, glucose, amino acids, and bicarbonate ^(2,84)^. Unlike other forms of HR, which primarily affect phosphate metabolism, Fanconi syndrome presents with a broader spectrum of metabolic derangements, including glycosuria, aminoaciduria, and non-gap metabolic acidosis. The condition may be congenital or acquired, with diverse etiologies including genetic, drug-in-duced, and systemic disorders.

Genetic causes of Fanconi syndrome present early in life, leading to progressive renal dysfunction, growth retardation, and metabolic bone disease. One well-characterized genetic form is Dent’s disease, an X-linked recessive disorder caused by loss-of-function variants in the *CLCN5* or *OCRL* genes ^(85)^. Both impair proximal tubular endosomal trafficking and protein reabsorption. Patients with Dent’s disease exhibit low molecular weight proteinuria, hypercalciuria, nephrocalcinosis and progressive renal failure, as well as phosphate wasting and rickets ^(86)^.

Cystinosis is another major genetic cause, an autosomal recessive lysosomal storage disorder caused by pathogenic variants in the *CTNS* gene, which encodes the lysosomal cystine transporter ^(87)^. Cystine accumulation within lysosomes lead to tubular damage and dysfunction, manifesting as Fanconi syndrome in infancy. Without treatment, cystinosis progresses to end-stage renal disease (ESRD) ^(88)^.

Acquired Fanconi syndrome can result from various environmental, pharmacological, and systemic insults that damage the proximal tubules. Drug-induced nephrotoxicity is a common etiology, specifically from agents like valproic acid, and expired tetracyclines. Also Tenofovir Disoproxil Fumarate (TDF), a common antiretroviral for HIV, has been strongly associated with proximal tubulopathy, characterized by phosphate wasting, proteinuria, and progressive kidney injury ^(89)^.

Heavy metal poisoning, specifically with lead, cadmium, or mercury, can induce proximal tubule dysfunction, leading to Fanconi syndrome. Chronic exposure to such metals is typically occupational or environmental, with long-term consequences including osteomalacia, anemia, and nephropathy ^(90)^.

Systemic conditions such as multiple myeloma ^(91)^, amyloidosis ^(92)^ and autoimmune disorders (like Sjögren’s syndrome) ^(93)^ can further contribute to acquired Fanconi syndrome. Such conditions may cause protein accumulation or inflammatory damage within renal tubules ^(94)^.

Patients with Fanconi syndrome exhibit profound metabolic disturbances, often presenting with polyuria, polydipsia, rickets, growth failure, and muscle weakness due to chronic electrolyte imbalances. A diagnostic workup includes serum biochemical markers, urinary studies, imaging, and bone studies.

Treatment of Fanconi syndrome focuses on correcting metabolic abnormalities and managing the underlying cause. Phosphate and bicarbonate supplementation are common therapies, often used in conjunction with specific treatments for genetic or acquired conditions ^(84)^. In cases of cystinosis, cysteamine therapy significantly delays renal decline ^(87)^. Withdrawal of nephrotoxic agents is essential for drug-induced cases ^(2)^.

### Emerging therapies and future directions for FGF23-independent HR

For disorders caused by impaired renal phosphate transport or proximal tubulopathies, therapeutic innovation is focusing on transporter modulation and replacement and strategies to mitigate the pathophysiology. Proof-of-concept studies of ENPP1-Fc enzyme replacement suggest benefit for ENPP1 deficiency-associated rickets and arterial calcification by restoring extracellular pyrophosphate levels ^(66,95)^, and analogous pyrophosphate-based strategies may be relevant for mitigating renal calcification in patient with HHRH, as suggested by experimental studies in NPT2a-deficient mouse models ^(96)^. Additionally, case reports indicate that fluconazole ^(97)^ or rifampin ^(98)^ may lower excessive 1,25(OH)_2_D and mitigate hypercalciuria in disorders characterized by excessive calcitriol, including *SLC34A3*-related HHRH. Advances in targeted delivery of gene therapies for *SLC34A3, CLCN5*, or *CTNS* could eventually enable curative strategies using specialized adeno-associated viruses ^(66)^ or CAR-T cells ^(99)^ to deliver the therapeutic. Progress in biomarker discovery and standardized functional assays will also be crucial to shorten the diagnostic pathway and align patients with the most appropriate therapy early in the disease course.

## HORMONAL ADAPTATIONS IN HYPOPHOSPHATEMIC DISORDERS

Accurate interpretation of serum levels of PTH, FGF23, 1, 25 dihydroxyvitamin D (1, 25(OH2)D) requires a clear understanding of expected hormonal adaptations. Recognizing typical patterns is critical for distinguishing primary disorders of parathyroid hormone excess, vitamin D deficiency, FGF23-mediated phosphate wasting, and intrinsic renal phosphate transport defects.

In primary hyperparathyroidism, elevated PTH drives renal phosphate wasting and increases calcitriol synthesis ^(13)^. Serum phosphate is typically low or low-normal, FGF23 may be secondarily elevated, and 1,25(OH)_2_D levels are often high-normal or elevated due to PTH stimulation of CYP27B1 and inhibition of CYP24A1 ^(44)^.

In secondary hyperparathyroidism due to vitamin D deficiency, low 25-(OH)D results in reduced calcitriol production, impaired intestinal calcium absorption, resulting in compensatory elevation of PTH. Serum phosphate may be low due to PTH-mediated phosphaturia, FGF23 levels are generally low or normal low, and 1,25(OH)_2_D is low or normal ^(37)^.

In FGF23-dependent hypophosphatemias such as XLH, excess intact FGF23 leads to renal phosphate wasting ^(6)^ and suppression of calcitriol synthesis ^(16)^. Serum phosphate is low, FGF23 is elevated, 1,25(OH)_2_D is low or normal, and PTH is typically normal or mildly elevated secondary to calcitriol deficiency and chronic phosphate therapy.

In FGF23-independent disorders, such as HHRH, renal phosphate wasting occurs in the setting of low or suppressed FGF23. Reduced phosphate and suppressed FGF23 stimulate increased calcitriol production, leading to elevated 1,25(OH)_2_D levels, enhanced intestinal calcium absorption, suppressed PTH, and hypercalcuria ^(83)^.

## DIAGNOSTIC APPROACH TO HYPOPHOSPHATEMIC RICKETS

The diagnostic evaluation of HR requires a comprehensive, stepwise approach to differentiate between intestinal and renal causes, which are further classified into FGF23-dependent and -independent renal causes. Due to the range of genetic, acquired, and metabolic disorders that can contribute to phosphate wasting, a structured assessment of biochemical markers, urinary studies, imaging findings, and genetic testing is required.

Measurement of circulating FGF23 plays a central role in differentiating FGF23-dependent from FGF23-independent hypophosphatemic disorders; however, interpretation requires understanding of available assay methodologies. Two main types of immunoassays are used in clinical practice: C-terminal assays and intact FGF23 assays. C-terminal assays detect both intact biologically active FGF23 and inactive or inhibitory C-terminal cleavage fragments, whereas intact assays specifically quantify the full-length active hormone ^(79)^.

In conditions such as iron deficiency or inflammation, FGF23 transcription may be upregulated with concurrent increased proteolytic cleavage ^(7)^. In these settings, C-terminal FGF23 levels may be elevated while intact FGF23 remains normal, potentially leading to diagnostic ambiguity. Conversely, in FGF23-dependent hypophosphatemic rickets (e.g., XLH, ADHR, TIO), intact FGF23 is inappropriately elevated relative to serum phosphate levels ^(6)^.

Assay standardization remains limited, and reference ranges differ between platforms, making interpretation assay-specific. Furthermore, FGF23 results should always be interpreted in conjunction with serum phosphate, calcitriol, urinary phosphate handling, and the clinical context.

Stepwise diagnostic evaluation

- Urinary studies
  - **Tubular reabsorption of phosphate (TRP):** calculated using paired fasting serum and second-morning void urine samples, whereas calcium excretion is typically determined from 24-hour urine collections when clinically indicated ^(18)^. Fasting samples are preferred to minimize postprandial variation. Urinary calcium excretion: elevated in FGF23-independent, normal in most FGF23-dependent HR.
  - **Urinary glucose, amino acids, β2-microglobulin:** elevated in Fanconi syndrome, indicating generalized proximal tubular dysfunction.
- Serum biochemical markers
  - **Serum phosphate:** low in all forms of HR.
  - **Serum calcium** is typically normal; however, it may be low in patients with FGF23-dependent HR ^(37)^.
  - **Calcitriol:** normal to low in FGF23-dependent rickets, elevated in FGF23-independent forms ^(16)^.
  - **PTH:** usually normal, may be elevated (secondary hyperparathyroidism) in FGF23-dependent HR.
  - **Serum FGF23:** elevated in FGF23-dependent HR, normal to low in FGF23-independent forms.
- Genetic testing
  - **Genetic panels** using whole exome or whole genome sequencing offered by commercial or academic providers now screen several known genes for pathogenic variants (see **Table 1**).

**Table 1:** Renal hypophosphatemic rickets: for clinical diagnostic purposes, these disorders are best classified into FGF23-dependent and FGF23-independent disorders

Disorder	Inheritance	Gene Defect	Serum Phosphate	FGF23	PTH	25-OHD	1,25 (OH)_2_D	Urinary Calcium	Key Features
Primary Hyperparathyroidism	Autosomal Dominant	*CAR, AP2S1, GNAS11*	↓ / low-normal	↑ / N	↑↑	N	↑	↑	Hypercalcemia, PTH-driven phosphaturia
Vitamin D Deficiency (Secondary HPT)	Autosomal Dominant or Recessive, acquired	*VDR, CYP27B1*	↓	↓ / N	↑	↓	↓ / N	↓ / N	Secondary hyperpara-thyroidism
XLH	X-linked dominant	*PHEX*	↓↓	↑↑	↑ / N	N	↓ / N	N	Dental abscesses, craniosynostosis
ADHR	Autosomal Dominant	*FGF23*	↓↓	↑ (CR)	↑ / N	N	↓ / N	N	Iron deficiency triggers onset
ARHR	Autosomal Recessive	*DMP1, ENPP1*	↓↓	↑	↑ / N	N	↓ / N	N	May overlap with GACI (*ENPP1*)
TIO	Sporadic, somatic	*FN1-FGFR1 / FN1-FGF1*	↓	↑	N	N	↓	N	Tumor localized by DOTATATE PET/CT
HHRH	Autosomal Recessive	*SLC34A3*	↓↓	↓ / N	↓	N	↑↑	↑↑	Hypercalciuria, kidney stones
Fanconi Syndrome	Autosomal Recessive, X-linked, acquired	*CTNS, CLCN5, OCRL*, etc.	↓	N	N	N	Variable	Variable	Multi-solute wasting

N: normal; GACI: generalized arterial calcification of infancy.
- Imaging studies
  - **Skeletal radiographs:** used in children to show characteristic signs of rickets (metaphyseal widening, fraying, bowing deformities).
  - **Dual-energy X-ray absorptiometry (DXA):** used in adults and adolescents with closed growth plates to quantify reduced bone mineral density (BMD).
  - **Functional imaging** (e.g., ^68^Ga-DOTATATE PET/CT) can identify the re-sponsible phosphaturic mesenchymal tumor in patients with TIO by detecting somatostatin receptor expression ^(69)^.

## CLINICAL IMPLICATIONS

In addition to maintaining a high degree of clinical vigilance to the possibility of HR in patients presenting with unexplained rickets, bone pain, fractures, short stature, or dental abnormalities, especially when serum phosphate is low and alkaline phosphatase is elevated, a stepwise approach that incorporates biochemical assessment, FGF23 quantification, imaging, and genetic testing is recommended ^(82)^. Treatment should be guided by etiology, patient age, symptom severity, and risk of complications. Regular follow-up and multidisciplinary management are key to achieving optimal outcomes and minimizing long-term effects.

## CONCLUSIONS

Hypophosphatemic rickets (HR) represents a diverse group of disorders unified by defective phosphate homeostasis, which ultimately impairs skeletal mineralization and leads to significant clinical consequences. The differentiation between FGF23-dependent and FGF23-independent etiologies is crucial in determining the optimal diagnostic and therapeutic approach, considering their distinct pathophysiological mechanisms, inheritance patterns, and treatment responses. Among FGF23-mediated disorders, X-linked hypophosphatemia (XLH) remains the most prevalent, while tumor-induced osteomalacia (TIO) is the most common acquired cause in adult-onset cases. Genetic defects in renal phosphate transporters characterize FGF23-independent forms, such as hereditary hypophosphatemic rickets with hypercalciuria (HHRH).

Early recognition and differentiation among HR subtypes are important for timely intervention, minimizing skeletal deformities, and improving patient quality of life. In addition to conventional therapy with phosphate ± active vitamin D analogs, the introduction of targeted therapies with burosumab has improved care in XLH and other FGF23-dependent forms of HR.

Despite these advances, several needs remain unmet. Standardized diagnostic algorithms, improved access to genetic screening, and long-term outcome data, particularly for novel therapies, such as fluconazole, rifampin, infigratinib, trametinib, and vosoritide, are necessary to optimize care. Research into gene editing technologies and FGF23 pathway modulators may hold curative potential for certain HR subtypes in the future. A collaborative effort across multiple clinical modalities is essential to advancing understanding and treatment of hypophosphatemic rickets.

## Data Availability

datasets related to this article will be available upon request to the corresponding author.
